# Can loss of agency and oppositional perturbation associated with antidepressant monotherapy and low-fidelity psychological treatment dilute the benefits of guideline-consistent depression treatment at the population level?

**DOI:** 10.1192/j.eurpsy.2020.86

**Published:** 2020-09-21

**Authors:** Johan Ormel, Fokko J. Bosker, Steven D. Hollon, Henricus G. Ruhe

**Affiliations:** 1 Department of Psychiatry, University of Groningen, University Medical Center Groningen, Groningen, The Netherlands; 2 Department of Psychiatry, University of Groningen, University Medical Center Groningen, Groningen, The Netherlands; 3 Department of Psychology, Vanderbilt University, Nashville, Tennessee, USA; 4 Department of Psychiatry, Radboudumc, Nijmegen, The Netherlands; 5 Donders Institute for Brain, Cognition and Behavior, Radboud University, Nijmegen, The Netherlands

**Keywords:** Depression, Treatment-Prevalence Paradox, Antidepressants, Psychological Treatment, Adverse effects

## Abstract

Despite major expansions of evidence-based treatments of common mental disorders in recent decades, especially antidepressant medication, the point prevalence of depression has not decreased; instead it probably increased in young adults. We question whether antidepressants (AD)-monotherapy and low-fidelity-to-guideline psychological treatment (PT) might have no effect or even adverse effects in some patients and contexts that dilute the benefits of treatment at the population level, making it harder for population-based studies to detect treatment-driven prevalence reductions. Randomized Clinical Trial (RCT)s have not identified these effects because AD-monotherapy and low-fidelity PT are uncommon in RCTs where treatment protocols are specified and carefully monitored, unlike treatment in real-world settings. Second, RCTs may have missed the bigger picture of ultimate outcomes due to too short follow-ups. We elaborate two mechanisms through which AD-monotherapy and low-fidelity PT could produce adverse effects on long-term illness course. Both mechanisms are speculative and we outline how to test.

Major depressive disorder is a common, highly heterogeneous disorder. To reduce its enormous burden [1], Western countries have expanded their mental health expenditures in specialty and primary care settings since the 1980s [2], providing more treatment for more people, primarily with antidepressants (AD) and, to a lesser extent, psychological treatments (PTs) [3,4]. Both Antidepressant (AD)s and PTs are well-established, evidence-based treatments with roughly similar modest short-term efficacy. Surprisingly, the point-prevalence of depression in the general population has not decreased since the wide-scale use of AD [5]; instead, the prevalence has probably increased, particularly in young people [6]. In addition, the prevalence of recurrent-chronic and treatment-resistant depression seems to have risen [7]. One obvious explanation for this “treatment-prevalence paradox” is increased first incidence, offsetting a treatment-driven prevalence drop. But population-based incidence studies do not find such an increase [4,8]. What else could explain the paradox?

## Explaining the Treatment-Prevalence Paradox

We question whether *AD-monotherapy and low-fidelity-to-guideline PT might have no effect or even adverse effects in some patients and contexts that dilute the benefits of treatment at the population level,* making it harder for population-based studies to detect treatment-driven prevalence reductions. The hypothesized mechanisms through which AD-monotherapy and low-fidelity PT could produce adverse effects on long-term illness course include Loss of Agency [9] and Oppositional Perturbation [10] or Tolerance [11,12]. Both mechanisms are speculative and need rigorous testing. RCTs have not identified these effects because AD-monotherapy and low-fidelity PT are uncommon in RCTs where treatment protocols are specified and carefully monitored, unlike treatment in real-world settings. In addition, AD-withdrawal studies may have missed the bigger picture of improved ultimate outcomes, due to misinterpreted withdrawal symptoms [13] and too short follow-ups as has been the case with antipsychotic withdrawal studies [14].

## Loss of Agency and Self-Help Activities

Loss of Agency refers to loss of self-efficacy, problem solving, and other self-help activities that normally benefit recovery [9]. Without treatment, depressed people often engage independently in self-help strategies, such as exercising, increasing pleasant activities, reducing stress, and meditation [15]. These self-help strategies can have two benefits. First, self-help activities can be therapeutic on their own. Second, these activities may empower people to believe in their own “agency” and “self-efficacy” for coping with depression and underlying problems. Successful experiences provide individuals with a greater sense of their own abilities, rather than feeling broken and dependent on others or medication to fix them [16]. For example, wait-list controls do worse than other untreated subjects, likely because, while awaiting treatment, people do not do the things that they might otherwise do to feel better [17]. If people on AD-monotherapy or low fidelity PT avoid or reduce self-help activities, the benefits of treatment may be more than offset by the loss of agency. Although exact data on the prevalence of AD-monotherapy and low fidelity PT are lacking, there is ample evidence of major treatment quality gaps [18].

According to the “network hypothesis” of depression, AD may act by enhancing neuronal plasticity, which allows environmental inputs to modify the neuronal networks to better fine tune the individual to the outside world [19]. Recent observations in the visual cortex directly support this idea [20]. This suggests that antidepressant drugs should not be used alone, but should be combined with interventions to guide the plastic networks within the brain by providing appropriate environmental input (e.g., behavioral activation and meditation).

The risk of Loss of Agency may be substantial for AD-monotherapy and low-fidelity PT provided without forms of empowerment. The counterproductive effects probably depend on provider characteristics, patient’s premorbid personality, and contextual factors. Nowadays, more than 80% of Selective Serotonin Reuptake Inhibitor (SSRI) prescriptions are written by General practitioners (GP)s, who may have fewer empowering strategies in their armamentarium or time to implement those. People in disadvantaged communities might thus be deprived twice over because they tend to receive more AD-monotherapy and less rigorous PT treatment compared to the more comprehensive service delivery of combined AD and empowering psychotherapy in affluent areas [9].

## Oppositional Perturbation and Symptom-Return

Oppositional perturbation refers to the AD-induced state of built-up perturbation in homeostatic monoamine regulatory mechanisms [10,12]. AD drive up the levels of neurotransmitters in the synapse. According to this theory, in brief, underlying homeostatic mechanisms are hypothesized to respond by shutting down synthesis presynaptically and reducing sensitivity post-synaptically, which establishes homeostatic regulation dependent on the ongoing intake of medication. Therefore, this process is expected to create a persistent state of perturbation. It has been proposed that, in a sense, AD “hijack” the homeostatic monoamine regulatory mechanisms. However, this AD-driven perturbation “bounces back” when AD are discontinued, and might overshoot the normal balance of monoamine storage and release, increasing the risk for symptom return compared to spontaneous remission. Importantly, direct evidence for oppositional perturbation is lacking, but the overshoot appears proportional to the extent that the class of AD perturbs the underlying neurotransmitter systems and corresponds with the likelihood of symptom return once AD are discontinued [10,12].

Some puzzling AD-related observations feed the idea of oppositional perturbation. First, the excess risk of symptom-return in remitted patients after AD discontinuation relative to that in remitted patients after Cognitive Behavioral Therapy (CBT) discontinuation [21] and AD continuation [22]. The excess risk has usually been interpreted as indicating that AD’s beneficial effects end at discontinuation. This interpretation is based on the plausible assumption that AD exposure is benign and has no lingering negative effects. Although to some extent, the observed excess risk may also be due to misinterpreted withdrawal symptoms being classified as relapse/recurrence [13], this is unlikely the whole story given that the excess risk in the placebo-substitution arm persists 3–6 months after discontinuation [22]. Oppositional perturbation suggests an alternative explanation: the as prophylactic interpreted effects of CBT and AD-continuation may be (partly) deceptive because instead there could be an increased risk of relapse/recurrence *due* to AD treatment (i.e., oppositional perturbation) that subsequent discontinuation unveils.

Second, some AD trials showed stepwise loss of effectiveness [23]. Bosman’s review of 10 studies examining failure to respond upon resumption of previously effective AD after a period of nonuse (tachyphylaxis) found that 16.5% of the 394 remitted patients who restarted AD after their symptoms returned experienced failure to respond (range 3.8–42.9%). Tachyphylaxis occurred in all AD-classes and has not been observed for CBT [24]. Although these nonresponders may alternatively be the patients who have had a placebo-response earlier, tachyphylaxis could also be due to oppositional perturbation with possible cumulative effects of agency loss, especially in patients on AD-monotherapy.

Third, risk of symptom-return during long-term AD is substantial. Multiple studies report 3-year cumulative risks exceeding 40% [25]. The risk is particularly high among patients with residual symptoms after acute treatment [26]. A variety of factors may drive symptom-return during maintenance AD, including suboptimal compliance and increased environmental stress. However, in addition, “loss of protection” in patients on AD-monotherapy (i.e., without empowerment), speculatively, might also reflect loss of agency and/or oppositional perturbation. Some authors even speculated that “tardive dysphoria” could develop in some predisposed individuals with prolonged AD-treatment [7].

## How to Investigate the Hypotheses

**Figure 1.**
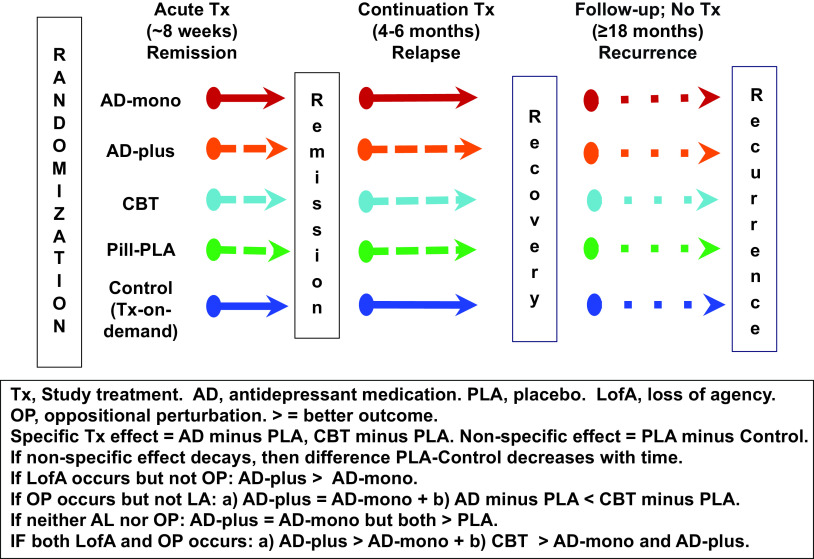
Schematic design of a prototypical RCT to test loss of agency and oppositional perturbation.

Given the aforementioned uncertainties, the rather alarming features of both loss of agency and oppositional perturbation, still without empirical investigation, urgently require research to quantify these effects. Acknowledging formidable feasibility and ethical issues (which cannot be elaborated here), the schematic RCT (Figure 1) enables their investigation. AD-free depressed individuals are randomized into five arms: “AD-monotherapy,” “AD-plus” (AD supplemented with an agency-enhancing/empowering component), “CBT,” “pill-placebo” (PLA), and Control (ethically acceptable treatment-on-demand group [27]). Patients in all arms are monitored for at least 2 years and anyone who does not show a minimum amount of improvement is considered to be “nonresponsive” and is pulled from the trial, while research assessments continue to monitor treatment and course. Later, AD discontinuation should be very gradual with careful assessments to reduce risk of misclassification of withdrawal symptoms as relapse/recurrence. The accompanying text in Figure 1 details the critical comparisons.

Comparing the long-term outcome of AD, CBT, and PLA is also informative because AD and CBT can innovatively be compared with the baseline of the PLA arm (lacking a specific AD or CBT treatment mechanism). This will address whether CBT truly has an enduring effect after discontinuation in patients remitted on CBT or merely appears so in comparison to possible adverse effects of AD after discontinuation in patients remitted on AD. The design also permits estimation of the magnitude of spontaneous remission (Control), nonspecific treatment effects (PLA vs. control), and treatment-specific effects (AD vs. PLA and CBT vs. PLA) in both short- and long-term outcomes.

## Conclusion

The hypothesized mechanisms of loss of agency and oppositional perturbation, together with AD-monotherapy and low-fidelity PT, might contribute to counterproductive effects on long-term illness course but need thorough empirical testing. If present, this would dilute the beneficial impact of guideline-consistent treatment at the population level, which could help explain the treatment-prevalence paradox. Given their public health potential, it is important to urgently investigate loss of agency and oppositional perturbation, which currently are alarming but so-far speculative hypotheses that have not been investigated.

## Data Availability

The data that support the findings, hypotheses and opinions of this Perspective have been published in the references cited in the paper.
